# The 12 items Amharic version WHODAS-2 showed cultural adaptation and used to measure disability among road traffic trauma victims in Ethiopia

**DOI:** 10.1186/s40359-020-00492-4

**Published:** 2021-01-02

**Authors:** Zewditu Abdissa Denu, Mensur Osman Yassin, Telake Azale Bisetegn, Gashaw Andargie Biks, Kassahun Alemu Gelaye

**Affiliations:** 1 Department of Anesthesia, School of Medicine, College of Medicine and Health Sciences, University of Gondar, Gondar, Ethiopia; 2 Department of Surgery, School of Medicine College of Medicine and Health Sciences, University of Gondar, Gondar, Ethiopia; 3 Department of Health Communication and Behavioral Science, Institute of Public Health, College of Medicine and Health Sciences, University of Gondar, Gondar, Ethiopia; 4 Department of Health Policy and Management, Institute of Public health, College of Medicine and Health Sciences, University of Gondar, Gondar, Ethiopia; 5 Department of Epidemiology and Biostatistics, Institute of Public Health, College of Medicine and Health Sciences, University of Gondar, Gondar, Ethiopia

**Keywords:** Validation, Dis-ability, Road traffic injury, Gondar, Amhara, Ethiopia

## Abstract

**Background:**

Adapting and translating already developed tools to different cultures is a complex process, but once done, it increases the validity of the construct to be measured. This study aimed to assess the 12 items WHODAS-2 and test its psychometric properties among road traffic injury victims in Ethiopia. This study aimed to translate the 12 items WHODAS- 2 interview-based tools into Amharic and examine the psychometric properties of the new version among road traffic injury victims.

**Methods:**

The 12 items WHODAS 2 was first translated into Amharic by two experts. Back translation was done by two English experts. A group of experts reviewed the forward and backward translation. A total of 240 patients with road traffic injury completed the questionnaires at three selected Hospitals in Amhara Regional State. Internal consistency was; assessed using Chronbach’s alpha, convergent, and divergent validity, which were; tested via factor analysis. Confirmatory factor analysis (CFA); was computed, and the model fit; was examined.

**Results:**

The translated Amharic version 12 –items WHODAS-2 showed that good cross-cultural adaptation and internal consistency (Chronbach’s α =0.88). The six factor structure best fits data (model fitness indices; CFI = 0.962, RMSEA = 0.042, RMR = 0.072, GFI = 0.961, chi-square value/degree of freedom = 1.42, TLI = 0.935 and PCLOSE = 0.68). Our analysis showed that from the six domains, mobility is the dominant factor explaining 95% of variability in disability.

**Conclusion:**

The 12 items interview-based Amharic version WHODAS-2; showed good cultural adaptation at three different settings of Amhara Regional State and can be used to measure dis-ability following a road traffic injury.

## Introduction

Injury is responsible for 16% of the global burden of diseases that disproportionately affects low and middle-income countries [1, 2]. According to the world health organization, 91% of injury-related deaths and 94% of disability-adjusted life years lost occurs in low and middle-income countries [3, 4].

Road traffic injuries, violence, falls, burns and assaults are the leading causes of disability [5]. Road traffic injuries are the leading causes of injury-related disability that is responsible for between 20 and 50 million peoples’ dis-ability globally. According to the world health organization report, 93% of this burden is disproportionately shared by low and middle-income countries having only 60% of the global registered vehicle [6]. Ethiopia is one of the countries in the Sub-Saharan regions that are seriously hit by road traffic injures [7].

Though there is a significant burden of Road traffic injury in the country, there is scarcity of evidence showing its magnitude and severity. Besides, the available studies so far were mainly showing the extent of mortality following road traffic injuries, whereas mortality is the only tip of a very large ice burg for the hidden burden of dis-ability following a road traffic injury. Lack of reliable and valid instruments could be one reason for the mentioned gap [8].

Disability is a collective term describing limitations in physical, mental, and social interaction or participation of a person following a disease condition or trauma [9]. It is challenging to quantify latent variables like disability directly but can be assessed indirectly from its domains [10]. Several ways of assessing disability; had been proposed by different scholars. Some of these tools include the functional independence measure (FIM) [11], the Nottingham Health Profile (NHP) [12], the London Handicaps Scale [13], the short form 36 (SF-36 form) [14], and Barthel’s index of activities (BAI) [15]. The WHO Disability Assessment Schedule (WHODAS-2) is one of the available tools that measure disability.

Among the available tools proposed to measure disability, we preferred WHODAS-2 because it incorporated the theoretical framework of dis-ability and had been tested; for its psychometrical properties among different groups and settings [16]. Besides, WHODAS-2; was entirely based on ICF (International classification of function); that incorporates physical, mental, and substance use disorders. It also assesses disability in a culturally sensitive way across a standard rating scale [10]. The tool measures disability from the perspective of six domains as perceived by an individual [17]. These domains include cognition, mobility, self-care, interpersonal relationships, activities of daily living, and participation in social interactions [18]. The tool has undergone different revisions since its development in 1998, by conducting several surveys among different cultures and languages to check for its validity and reliability and found to be psychometrically robust [19, 20].

WHODAS − 2; was first developed through 10 years of collaborative work of scientists around the world. The tool; was initially designed to assess disability among psychiatric patients [21]. Later on, it was revised to measure disability from different causes incorporating different aspects of health [22]. The initial WHODAS-2 had 96 items under six domains; that was very long and required an interview time of 63–94 min [16]. Revision of this tool; was done by conducting a field survey at different countries by administering the questionnaires concurrently with other scales, such as the Medical Outcome Study, 12-item Short-Form Survey (SF12), the SF-36, the London Handicap Scale (LHS), WHOQOL (World Health Organization Quality of Life) or the WHOQOL-BREF. The survey; was conducted in different countries from which 34 items; were developed. Finally, the full version WHODAS − 2 was developed; by adding two items from feedback collected [23]. WHODAS-2 is available in three versions (a 36-item, 12-item, and 12 + 24-item version).

The 12 items WHODAS − 2 has been derived from the 36-item version to provide a briefer tool for assessing overall functioning in surveys or health outcome studies [16]. It had been confirmed to have good reliability and had been; reported to explain 81% of the variability observed in the full version WHODAS-2 [16].

WHODAS-2: which is a generic tool is; non-disease specific, but its validity, reliability, and responsiveness; had been tested among different clinical conditions including, patients with chronic illnesses [24], multiple Sclerosis [25], ankylosing spondylitis [26], musculoskeletal pain [27], mothers with severe maternal morbidity [28], altered functioning during the postnatal period [29], disabilities caused by different neoplastic disorders [30, 31], motor disabilities [32], patients with depression and back pain and patients with severe mental illnesses [33, 34]. In all the mentioned studies, WHODAS-2; had shown internal consistency and validity with the underlining clinical conditions of participants’ or other tools measuring disability.

WHODAS- 2 had; also been used in the evaluation of the effectiveness of interventions for different disabling conditions including, the assessment of outcomes of dementia, comparing those in the community with those with long term therapy [35], evaluation of community- based interventions in reducing disability among schizophrenic patients [36], evaluation of interventions for patients with depressive disorders [37], evaluation of the effectiveness of hearing loss interventions [38], the success of surgical interventions for different ranges of clinical conditions [39] and assessment of the effectiveness of primary care in reducing disability in depression and back pain [33].

Though WHODAS 2.0; had been used in a wide range of health conditions, its repeatability; had not been tested among Amharic speaking trauma victims. Having a standardized and valid tool is an essential step in quantifying the burden of any health condition. Cross-cultural adaptation of the available tools is vital as many of these tools; were developed in high-income countries that are assumed to have different cultures, social interactions, and life activities from low-income countries [40].

Validating a tool developed somewhere else; is essential as the way of life activities and ways of expressing emotions and social interactions are different among different cultures. Freely stated terms in one culture; could be a taboo word in other settings [41]. Besides, using a valid and standardized tool than developing a local tool will ensure the comparability of the finding across different cultures [42].

The aim of the current study was, therefore, to evaluate the psychometric properties of the Amharic translated version of the brief WHODAS-2.0 among road traffic injury victims in Northwest Ethiopia. The developed tool would be used by future researchers to quantify the burden of disability following trauma such as road traffic injuries.

## Method

The full version WHODS-2 has 36 items under six domains. This version has six items under the cognition domain, five items under mobility, four items under self-care, five items under getting along, and the remaining two Domains; life activity and community participation; contain eight items each [43]. The 36 items version was shorter than the 96 items, and it takes 20 min for the interview [44].

The 12 item WHODAS II has three versions based on means of administration as interview-based, self-administered, and proxy administered. This version was; found to explain 81% of the variability of the 36 items WHODAS II [45].

### Data collection tools

We measured functional impairment using 12 items WHODAS-2 having; six domains that are reported on five points Likert scale from 0 to 4 based on the severity of the problem. 0 = no difficulty, 1 = mild difficulty, 2 = moderate difficulty, = severe difficulty, 4 = very severe difficulty. The minimum score was 0, and the maximum is 48. Socio-demographic variables such as age, sex, residence, educational status, and occupation were collected using structured questions. Injury-related variables were; collected from the victims’ medical charts.

### Translation and adaptation of 12 items WHODAS 2 into Amharic

From the available WHODAS-2 tools, we selected the 12 items interview-based version to translate and adapt it in the context of our community. This version was; chosen because it is brief and can be administered within a short period that makes it suitable to be used in clinical setup for assessment of functional impairments. We preferred the interview-based version as our participants include both literate and illiterate people. Forward and backward translations were done by four university instructors who were urgent Amharic speakers, trained at masters’ degree level, and had; experiences of conducting different researches in the area of public health. The translated version was; then presented to a panel of experts who were, selected based on their field of experts. All the experts were members of the University of Gondar.

The expert committee constituted a clinical psychologist and public health practitioners trained at the Ph.D. level, three physiotherapists trained at masters’ degree level, and one language expert. The experts checked whether the meanings of the original items were; not altered and evaluated whether the items were measuring the same concept. The expert committee critically evaluated each “item” for semantic and idiomatic equivalence and re-phrased some words to be more understandable; without losing the original meaning and concept. Semantic and idiomatic equivalence of the back translation was checked by another expert who has a Ph.D. degree in public health and urgent in the English language, and a native speaker of the target language.

**Fig. 1:**
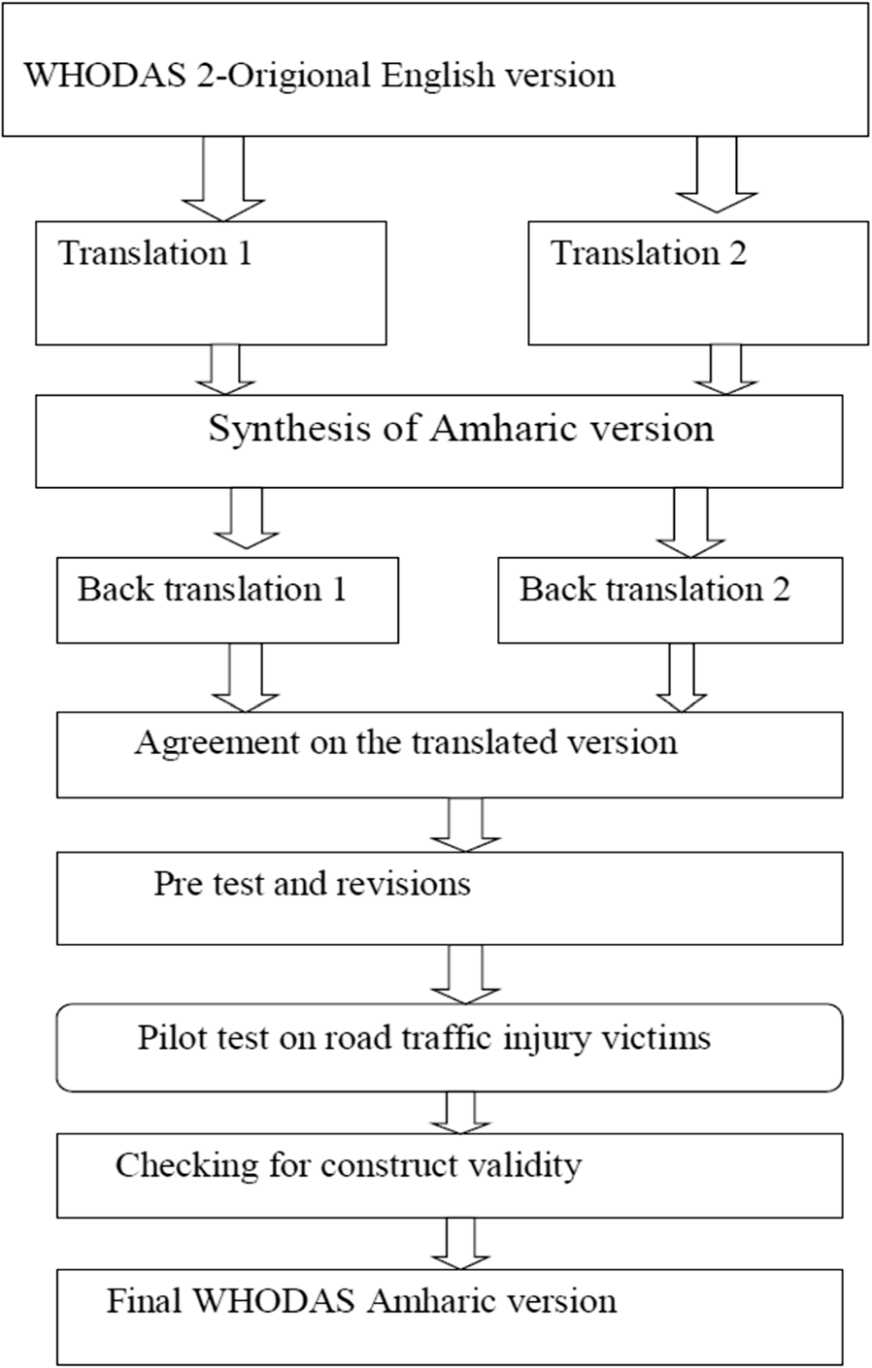
Translation process

After incorporating suggested corrections and comments given by experts, the second Amharic version was; produced. The Amharic version; was then pretested on 12 road traffic injury victims who were, attending a follow-up clinic at the University of Gondar specialized Hospital. An in-depth interview was; conducted by a trained interviewer who is a naive Amharic speaker. Further revision of the tool was; done incorporating the comments; given by participants by asking their opinion on the locally acceptable words for some items. The participants’ age range was from 22 to 60 years. The mean age was 34.7 ± 13 (Fig. 1 ; Table 1).

**Table 1:** Socio-demographic characteristics of participants for the pre- test *n* = 12

Variables	Frequency	Percentage
Sex		
Male	9	75
Female	3	25
Age, mean with SD		
347 ± 13		
Educational status		
Can’t read and write	5	41.7
Can read and write only	2	16.7
Primary education	4	33.3
Secondary education	1	8.30
Tertiary education	0	0
Occupation		
Farmers	5	41.7
Government employee	3	25
Others	4	33.3
Residence		
Urban	7	58.3
Rural	5	41.7
Type of injury		
Mild	3	25
Moderate	3	25
Severe	6	50

### Phase 2: psychometric validation of the Amharic WHODAS 2 questionnaire

#### Study participants

This study was; conducted between March and June 2019 at the University of Gondar specialized Hospital, Tibebe Gihon, and Felege Hiwot referral Hospitals. Data were; collected from 240 road traffic injury victims attending an orthopedic clinic at both hospitals during the study period. The sample size was; based on the recommendation for factor analysis to have a minimum of 10–20 cases /item [46]. To maximize the model fit, we took 20 participants per item; hence, we included 240 study participants in this study. Cases here were our respondents (road traffic injury victims), and the number of “items” refers to questions under each domain/factor (total of 12 items).

### Data collection

Data were; collected from three hospitals in Amhara Regional State. The two hospitals (Felege Hiwot and Tibebe Gihon); are located in the capital city of Amhara Regional State, Bahirdar. Hundred fifty-seven consecutive cases were; collected; from Bahirdar, and the remaining 83 were; included from the University of Gondar Specialized Hospital. The inclusion criterion was all Adult road traffic injury victims visiting the orthopedic clinic with a minimum duration of injury of 1 month. Data were; collected by three trained BSc nurses working at orthopedic clinics of the respective hospitals using a face-to-face interview.

### Statistical analysis

The data were; analyzed using the statistical software package IBM SPSS Statistics version 23 with AMOS (Analysis of Moment Structures) extension. Socio-demographic characteristics of participants were; described using descriptive statistics. Content validity was; assessed using the content validity index (CVI) based on the evaluation of 6 experts in the fields of psychology, public health, and physiotherapy. The item content validity was; calculated using a four-value Likert scale ranging from 1 to 4 (representing irrelevant to very relevant). According to the literature, a content validity index of 78 and above is acceptable when the numbers of experts are greater or equal to six [47]. We assessed acceptability qualitatively by evaluating the understandability and acceptability of the items. Also; the time required to finish each interview [48].

### Construct validity

Construct validity is; defined as the degree to which an instrument measures the trait or theoretical construct that it is; intended to measure. Construct validity can be; evaluated using exploratory or Confirmatory Factor Analysis (CFA) [49, 50]. Exploratory factor analysis is; done when a researcher wants to know the pattern of responses. In such cases, the structure of factors will be data-driven; whereas, confirmatory factor analysis starts with a hypothesis about how many factors there are and which items load on which factor [51]. Since disability has a known dimension, we conducted confirmatory factor analysis.

### Confirmatory factor analysis (CFA)

CFA is a statistical technique used to verify the factor structure of a set of observed variables. CFA allows the researcher to test the hypothesis that a relationship between observed variables and their underlying latent construct exists [51, 52]. In confirmatory factor analysis, there is no way to improve the model fit by adding a regression line, but the goodness of fit of the model can be; improved by performing modifications as suggested by the software to put more covariance [53].

CFA was; performed using a generalized least square estimate, as our responses have an ordered categorical nature [54]. Factorability and adequacy of the sample were checked by conducting Kaiser– Meyer–Olkin test (KMO) > 0.5 and Bartlett’s Test of Sphericity (*p* < 0.05) [55, 56]. The goodness of fit indices were tested using Tucker Lewis Index (TLI; > 0.90 acceptable, > 0.95 excellent), the Comparative Fit Index (CFI; > 0.90 acceptable, > 0.95 excellent), and Root Mean Square Error of Approximation (RMSEA; < 0.08 acceptable, < 0.05 excellent), and Standardized Root Mean Residual (SRMR; < 0.08 acceptable) [57].

### Internal consistency

The internal consistency of the tool was; demonstrated using Cronbach’s α coefficient for each factor and the whole instrument. Composite reliability (CR) was; assessed using confirmatory factor analysis. Any value above 0.7 is considered as evidence of internal consistency [58]. Internal consistency; was also tested through the analysis of the correlations of items under each WHODAS- 2 domains/factors using confirmatory factor analysis (CFA) (CFA). The average variance extracted AVE), the maximum shared variance; (MSV), and composite reliability were; performed to evaluate whether the items listed under each domain/factor were; measuring the same thing. An average variance estimated (AVE) value of > 0.5 and composite reliability (CR > 0.7) are evidence that items measuring similar constraints were; loaded to one domain/factor [54, 59]; whereas; AVE > MSV (Maximum Shared Variance) is evidence for divergent validity [60].

### Concurrent validity

Construct validity; was assessed by comparing the WHODAS − 2 score with the injury severity score. The injury severity score was; calculated using the revised injury severity score that; based on anatomic body regions affected. In this scale, the three severely injured body regions have their score; squared and added together to produce the ISS score [59].

## Results

Socio-demographic characteristics of participants (*n* = 240) A total of 240 road traffic injury survivors attending a clinical follow-up at the selected hospitals were; included in this study. Of the total interviewed, 166 (69.2%) male and 74 (30.8%) females; were participated in the current study. The mean age was 33.5 ± 11.5 years. The minimum and maximum ages were 18 and 78 years, respectively (Table 2). As to injury severity, more than half of the participants had a severe injury that involved multiple sites (Table 3).

**Table 2:** Socio-demographic characteristics of study participants (*n* = 240)

Variables	Frequency	Percentage
Sex		
Male	166	69.2
Female	74	30.2
Age group		
18–30	115	47.9
31–50	105	43.8
≥ 51	20	8.3
Educational status		
Can’t read and write	74	30.8
Can read and write only	67	27.9
Primary education	68	28.8
Secondary education	21	8.8
Tertiary education	10	4.2
Occupation		
Farmers	116	48.3
House wives	40	16.7
Self-employee	60	25
Gov. employee	15	6.3
Others	9	3.8
Residence		
Urban	103	42.9
Rural	137	57.1
Site of injury		
Head & neck	28	11.7
Face	48	20.0
Chest	18	7.5
Abdomen	16	6.7
Extremity	50	20.8
Multiple organ	80	33.3

**Table 3:** Site of injury and injury severity score among participants n = 240

Variable	Frequency	Percentage
Site of injury		
Head & neck	28	11.7
Face	48	20.0
Chest	18	7.5
Abdomen	16	6.7
Extremity	50	20.8
Multiple organ	80	33.3
Injury severity		
Mild injury	54	22.5
Moderate injury	59	24.5
Severe injury	123	51.2
Very severe injury	4	1.7

The overall mean disability score was 22.6 ± 9.25. The lowest mean disability score was; observed in domain 4, item 2 (getting along with people) (0.7), and the highest mean disability score was; observed in D5, “item” 1 (life activity) with, a mean disability score of 2.9 (Table 4).

**Table 4:** Mean score and standard deviation for each of the WHODAS 2.0 items

items	Number	Mean score	SD
Concentrating on doing something for 10 minutes?	240	1.7167	1.13656
Learning a new task, for example, learning how to get to a new place?	240	1.8167	1.11275
Standing for long periods such as 30 min?	240	2.1125	1.44344
Walking a long distance such as a km or equivalent?	240	2.6958	1.57756
Washing your whole body?	240	1.7042	1.25761
Getting dressed?	240	1.8000	1.28134
Dealing with people you do not know?	240	.8875	.93729
Maintaining a friendship?	240	.7083	.94090
Taking care of your day to day activity	240	2.9000	1.39275
Your day-to-day work/school?	240	2.5875	1.32274
How much of a problem did you have in joining in community activities	240	2.6917	1.36787
How much have you been emotionally affected by your health problems?	240	2.5917	1.28393
Overall score	240	22.66	9.25

In general, the process of translation and adaptation of the WHODAS-2 into the Amharic version was satisfactory. The 12 item WHODAS-2 is the simplified version and has no sensitive words or taboo words in it. The expert panel examined the words used in the Amharic version for any taboo word and agreed that all the terms used; were culturally accepted.

But, some items were; found to be difficult; to be understood by our participants. We made slight modifications without altering the original meaning. Such difficulty was; observed in items under domain 1 (understanding and communicating). This difficulty was; solved by providing examples that can elaborate on the terms. The majority of our participants had; difficulty in understanding the item “Concentrating on doing something for 10 minutes”. This phrase was modified as “ability to perform tasks with concentration “in Amharic as “  ” Under domain 2; again majority of participants; had confused in responding to an item that states “walking a kilometer distance”. We solved by giving examples such as “ability to go to church or mosque every morning and evening” as such activity is; considered as a simple task expected to be done by a person who can walk in our culture, especially among the rural community.

The other common problem for almost all our participants was the statement that inquires the 30 days memory of difficulty of performing tasks. The responses were generally inconsistent with each other; therefore, this part was; excluded from the analysis. The interview was very smooth and without difficulty in the rest of the items.

### Internal consistency

Cronbach’s alpha for the 12 item WHODAS − 2 scale was 0.88 (CI 0.85–0.90). The correlation between items of the six domain ranges from 0.75 (self-care) to 0.96 (cognition). Cronbach’s alpha was 0.75 for self-care (CI 0.679–0.807), 0.917 for mobility (CI 0.892–0.935), 0.89 for life activities (CI 0.868–0.921), 0.960 for cognition (CI 0.948–0.969), 0.76 for getting along (CI 0.767–0.819), and 0.942 for participation (CI 0.948–0.969). (Table 5).

**Table 5:** Correlation coefficient between items of WHODAS-2 domains

Domains	Cronbach’s α with 95% CI
Cognition	0.96 (0.948–.969)
Mobility	0.91 (0.892–0.935)
Self- care	0.75 (0.679–0.807)
Getting along	0.76 (0.767–0.819)
Life activity	0.89 (0.868–0.921)
Participation	0.94 (0.948–0.969)
Overall	0.88 (0.85–0.90)

### Convergent validity

The construct validity was; tested through the analysis of the correlations of items under each domain/factor of WHODAS-2 (Table 5). The sample adequacy (KMO); was 0.757 that> 0.5 indicating, the sample was adequate. We computed the average variance estimated by taking a standardized variance estimate and divide it by the number of items under each domain/factor (sum square factor loadings/number of “items”). The AVE (average variance estimated) value for our Amharic version WHODAS-2 ranged from o.595 to 0.92 indicating, that there is; evidence for convergent validity (Table 6).

**Table 6:** Average variance estimated and maximum shared variance based on CFA

Factors	Standardized item loadings	Squared loadings	AVE	MSV
**F1**	0.988	0.976	0.924	0.370
	0.934	0.872		
**F2**	0.916	0.839	0.874	0.465
	0.954	0.910		
**F3**	0.771	0.594	0.595	0.203
	0.773	0.597		
**F4**	0.593	0.351	0.753	0.538
	1.075	1.155		
**F5**	0.999	0.998	0.832	0.451
	0.817	0.667		
**F6**	1.002	1.004	0.900	0.358
	0.893	0.797		

### Concurrent validity

Concurrent validity was; evaluated by computing the correlation between the WHODAS-2 score and injury severity scale. All the domains of WHODAS-2 and injury severity scale have a positive correlation. The correlation coefficient ranged from 0.39 to 0.84. The overall WHODAS-2; is strongly correlated with the injury severity score (Cronbach’s α =0.961) (Table 7).

**Table 7:** Correlation between injury severity score and Domains of WHODAS-2

Ser. no	WODAS-2 items	Cronbach’s α
**1.**	Concentrating on doing something	0.65
**2.**	Learning a new task, for example, learning how to get to a new place?	0.76
**3.**	Standing for long periods such as 30 min?	0.76
**4.**	Walking a long distance such as a km or equivalent?	0.84
**5.**	Washing your whole body?	0.65
**6.**	Getting dressed?	0.66
**7.**	Dealing with people you do not know?	0.39
**8.**	Maintaining a friendship?	0.41
**9.**	Taking care of your day to day activity	0.82
**10.**	Your day-to-day work/school?	0.75
**11.**	How much of a problem did you have in joining in community activities	0.79
**12.**	How much have you been emotionally affected by your health problems?	0.76
**13.**	Overall WHODAS-2 score	0.96

**Fig. 2:**
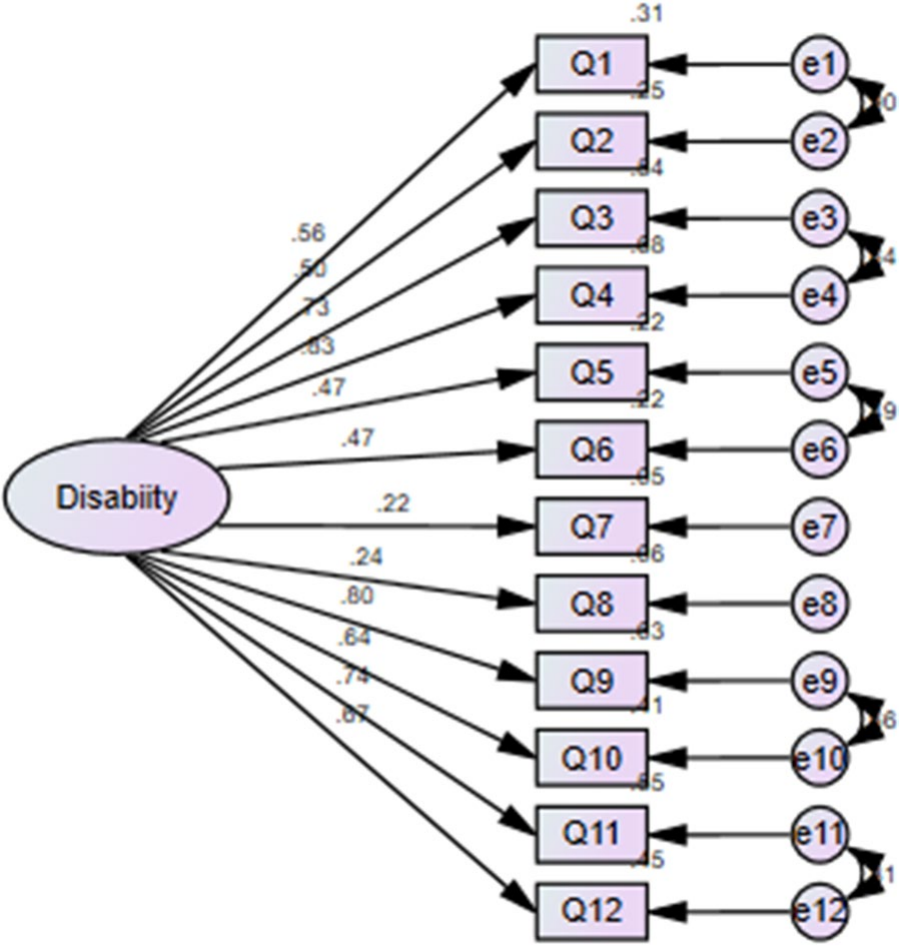
First order Confirmatory Factor stracture

**Fig. 3:**
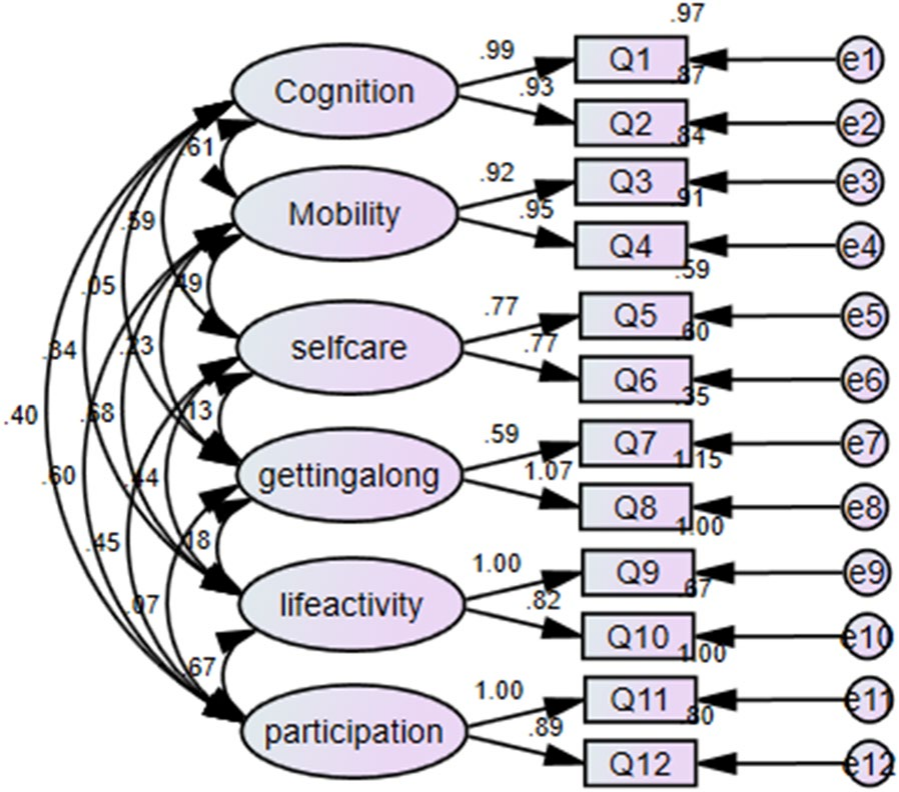
A six factor structure confirmatory factor analysis

We conducted confirmatory factor analysis using both 1-factor structure (Fig. 2) and six-factor structure (Fig. 3). The one-factor structure poorly fits with our data (χ2/df = 4.83; CFI = 0.92; TLI = 0.882, GFI = 0.878 and RMSEA = 0.127; RMR = 0.114; PCLOSE = 0.00) while the six-factor structure; best fits with our data based on the model fitness Indies (Fig. 3).

Our data indicated that a 6-factor structure fits the 12 items WHODAS 2.0 well. The factor loading ranges from 0.59 (getting along) to .95 (mobility) (Fig. 3). The goodness of t indices were within the acceptable ranges (χ2/df = 1.42; CFI = 0.963; TLI = 0.935, GFI = 0.962 and RMSEA = 0.042; RMR = 0.072; PCLOSE = 0.680) (Table 8). To assess how well the model matches the observed data, we used the “RMSEA” error of approximation as a primary indicator.

**Table 8:** Goodness of fit indices for confirmatory factor analysis (CFA)

Measure	Value	Threshold
Chi-square/df	55.5/39	< 3; < 5 sometimes permissible
*P* Value for the model	0.04	> 0.05
RMR	0.072	< 0.09
GFI	0.961	> 0.95
CFI	0.962	> 0.95
RMSEA	0.042	< 0.05
PCLOSE	0.680	> 0.05

## Discussion

The 12 items WHODAS-2 scale was; successfully translated and culturally adapted to Amharic. The study confirmed that the proposed 6 factor WHODAS2 structure had shown good metric properties among road traffic injury victims in Amhara regional state. The six-factor model most accurately fits the observed data. The finding is consistent with previous studies [61, 62]. But the result is inconsistent with previous studies that showed the brief version WHODAS-2 does fit best with the second-order factor structure or the unidirectional model proposed by WHO [26, 33, 63–65].

Generally, the Amharic brief version of WHODAS-2 is understandable by most of our participants. The slight difficulty was; observed in few items for which slight modifications and verifications with examples were; made while keeping the meaning of the original English version. “The cognitive “domain” was the most difficult to understand by our participants. A similar difficulty was; reported in the Korean study [66], but the finding is inconsistent with a study conducted among the rural community in Ethiopia that indicated almost all items were straightforward [33].

In addition to the cognitive Domain, slight confusion; was observed in the life activity domain, especially among male participants. This confusion is because a household activity is; a task given to women customarily. We tried to solve this problem by providing that can be; performed by both sexes at home. For our rural dwellers’ routine household activities are usually cooking and activities related to it. Therefore, we specified this by giving examples that can be; performed by men according to our culture such as, pasturage, leading the house as a whole, and financial management. A similar adaptation was; done by a previous study [67].

Concerning the scores of WHODAS − 2 domains, the highest score (a most challenging task) was in the “Life activities” domain, at work, as well as in the mobility domain, both standing and walking for 30 min. This finding is; consistent with previous studies [68, 69]. Two authors also reported, “mobility” to be the “domain” with the highest score among participants with functional limitations [70, 71]. This finding could be; explained by the fact that people with injury are more likely to have restrictions in life activities and mobility. The least difficulty score was; observed in the “getting along with people domain”.

The study showed that participants with higher injury severity scores had the highest WHODAS 2 scores. The overall correlation coefficient between injury severity score and WHODAS-2 score in the current study was 0.96. This finding is consistent with previous studies [65, 72] that reported a correlation coefficient of above 0.7.

The reliability test also indicated that the Amharic version WHODAS-2 scale can be; reproduced and valid to assess disability among road traffic injury victims in Amhara regional state. The Cronbach’s alpha value for the total scale was .88 (excellent internal consistency). The finding is consistent with studies [26, 33, 65, 73]; that showed WHODAS − 2 had “excellent” internal reliability with Chronbach’s α value of above 0.8.

The correlation between items of each “domain” ranges from 0.75 (self-care) to 0.96 (Cognition): showing there is evidence for internal consistency. Similar findings were; reported by previous studies [16, 74–76]. But V. Steinerte and colleagues reported; that the least correlation was; observed between items of communication domain [68]. Convergent validity is; ensured when “items” under a specific domain/factor correlate to each. Convergent validity is; claimed if the correlation coefficient is above 0.50 [74].

Our data demonstrated that the WHODAS-2 domains are; positively correlated with injury severity score that is evidence for convergent validity [74]. The least correlation was; observed between getting along and injury severity scale (Cronbach’s α = 0.39), and the highest correlation was between mobility and trauma severity score (Cronbach’s α = 0.84) (Table 7). This finding could be because trauma victims are more prone to physical injury that impairs mobility than getting along with people. Our result is consistent with [65] that showed victims with more severe injury levels had higher WHODAS − 2 score.

Confirmatory factor analysis confirmed a six-factor structure for the schedule with acceptable goodness of fit indices. The result is consistent with previous studies by [33, 77].

### Limitation of the study

Test-retest validity was; not carried out, so that sensitivity to change or treatment was; not tested; due to the feasibility issue. The study only included participants above 18 years, and we recommend future studies to test the validity of this instrument among adolescents that are also vulnerable to road traffic injury. Responsiveness overtime was; not assessed as we took data only at a point in time (cross-sectional design).

## Conclusion and recommendation

WHODAS-2; is successfully translated and culturally adapted into the Amharic version. Our study confirmed the validity, reliability, and factor structure of the 12 item WHODAS-2. Further research is; recommended to test for the responsiveness of the tool with better design.

## Data Availability

The datasets used and/or analyzed during the current study are available from the corresponding author on reasonable request.

## Abbreviations

AMOS: Analysis of Moment Structures
AVE: Average Variance Extracted
BAI: Barthel’s Index of activities
CFA: Confirmatory Factor Analysis
CFI: Comparative Fit Index
CR: Composite Reliability
CVI: Content Validity Index
FIM: Functional Independence Measure
GFI: Goodness of Fit Index
KMO: Kaiser – Meyer – Otkin Test
LHS: London Handicaps Scale
MSV: Maximum Shared Variance
NPH: Nottingham Health Profile
OPD: Outpatient Department
RMSEA: Root Mean Square Error of Approximation
SRMR: Standardized Root Mean Residual
TLI: Tucker Lewis Index
WHODAS-2: World Health Organization Disability Assessment Schedule
WHOQOL BREF: World Health Organization Quality of Life
